# Transcriptome-Based Molecular Networks Uncovered Interplay Between Druggable Genes of CD8^+^ T Cells and Changes in Immune Cell Landscape in Patients With Pulmonary Tuberculosis

**DOI:** 10.3389/fmed.2021.812857

**Published:** 2022-02-07

**Authors:** Faten Ahmad Alsulaimany, Nidal M. Omer Zabermawi, Haifa Almukadi, Snijesh V. Parambath, Preetha Jayasheela Shetty, Venkatesh Vaidyanathan, Ramu Elango, Babajan Babanaganapalli, Noor Ahmad Shaik

**Affiliations:** ^1^Department of Biology, Faculty of Sciences, King Abdulaziz University, Jeddah, Saudi Arabia; ^2^Department of Pharmacology and Toxicology, Faculty of Pharmacy, King Abdulaziz University, Jeddah, Saudi Arabia; ^3^Division of Molecular Medicine, St. John's Research Institute, Bangalore, India; ^4^Department of Biomedical Sciences, College of Medicine, Gulf Medical University, Ajman, United Arab Emirates; ^5^Auckland Cancer Society Research Centre (ACSRC), Faculty of Medical and Health Sciences (FM&HS), The University of Auckland, Auckland, New Zealand; ^6^Princess Al-Jawhara Al-Brahim Center of Excellence in Research of Hereditary Disorders, King Abdulaziz University, Jeddah, Saudi Arabia; ^7^Department of Genetic Medicine, Faculty of Medicine, King Abdulaziz University, Jeddah, Saudi Arabia

**Keywords:** *Mycobacterium tuberculosis*, gene express profile, drug target, CD8^+^T cells, immune pathways

## Abstract

**Background:**

Tuberculosis (TB) is a major infectious disease, where incomplete information about host genetics and immune responses is hindering the development of transformative therapies. This study characterized the immune cell landscape and blood transcriptomic profile of patients with pulmonary TB (PTB) to identify the potential therapeutic biomarkers.

**Methods:**

The blood transcriptome profile of patients with PTB and controls were used for fractionating immune cell populations with the CIBERSORT algorithm and then to identify differentially expressed genes (DEGs) with R/Bioconductor packages. Later, systems biology investigations (such as semantic similarity, gene correlation, and graph theory parameters) were implemented to prioritize druggable genes contributing to the immune cell alterations in patients with TB. Finally, real time-PCR (RT-PCR) was used to confirm gene expression levels.

**Results:**

Patients with PTB had higher levels of four immune subpopulations like CD8^+^ T cells (*P* = 1.9 × 10^−8^), natural killer (NK) cells resting (*P* = 6.3 × 10^−5^), monocytes (*P* = 6.4 × 10^−6^), and neutrophils (*P* = 1.6 × 10^−7^). The functional enrichment of 624 DEGs identified in the blood transcriptome of patients with PTB revealed major dysregulation of T cell-related ontologies and pathways (q ≤ 0.05). Of the 96 DEGs shared between transcriptome and immune cell types, 39 overlapped with TB meta-profiling genetic signatures, and their semantic similarity analysis with the remaining 57 genes, yielded 45 new candidate TB markers. This study identified 9 CD8^+^ T cell-associated genes (*ITK, CD2, CD6, CD247, ZAP70, CD3D, SH2D1A, CD3E*, and *IL7R*) as potential therapeutic targets of PTB by combining computational druggability and co-expression (r^2^ ≥ |0.7|) approaches.

**Conclusion:**

The changes in immune cell proportion and the downregulation of T cell-related genes may provide new insights in developing therapeutic compounds against chronic TB.

## Introduction

Tuberculosis (TB) is a chronic infectious lung disease caused by pathogenic *Mycobacterium tuberculosis (MTB)* belonging to *the Mycobacteriaceae* family. Despite the widespread use of antibiotics and live attenuated vaccine, TB remains to be the major cause of morbidity and death among all bacterial diseases (1). This is primarily due to the rapid emergence of drug-resistant *MTB* strains and the incomplete knowledge of complex host-pathogen interactions (2). In the initial stages of infection, *MTB* invades and replicates in the macrophages after reaching the alveolar air sacs of the lungs (3). Granulomas, hallmark of TB, are formed around the infected macrophages by the organized aggregation of immune cells (like T and B lymphocytes), multinucleated giant cells, dendritic cells, and fibroblasts. Granulomas also suppress the host immune responses, as dendritic cells and macrophages were unable to present antigen to lymphocytes (4). It is noteworthy to mention that mycobacteria can induce distinct host responses from asymptomatic conditions to severe pulmonary illness (5). However, underlying immune cell types and their association with the differentially expressed genes in TB and how they contribute to severe infection are not yet fully explored.

Over the past few decades, microarray-based genome-wide RNA profiling has evolved as a powerful approach to investigate the host transcriptional response (of ~19,000 genes) in infectious diseases (6). However, differences in the type of clinical samples, array platforms, and statistical approaches used, created a discordance in interpreting massive transcriptomics data. Advances in statistical modeling and bioinformatics approaches have accelerated the identification of disease-centric genes by employing gene networking methods based on graph topological parameters for many infectious diseases (7, 8). Moreover, the new bioinformatic methods like estimating relative subsets of RNA transcripts (CIBERSORT), Tumor Immune Estimation Resource (TIMER), and Estimating the Proportions of Immune and Cancer cells (EPIC) are developed to characterize immune cell composition using large-scale gene expression data (9, 10). These bioinformatic methods implement functional enrichment scores based on the presence of the query genes over reference gene sets. They perform variety of biological analyses including immune responses based on the defined gene sets. Exploring abnormal immune cell infiltration is critical for developing novel transformative therapies to combat diseases such as cancer, myocarditis, and TB (11, 12). Therefore, in order to characterize alterations in immune cell proportion landscape and transcriptomic profile, and to identify new molecular therapeutic targets, this study applied statistical and knowledge-based systemic investigations (such as semantic similarity, gene correlation, and graph theory parameters) to the blood transcription data of patients with TB.

## Materials and Methods

### Study Design and Global Expression Data

**Figure 1:**
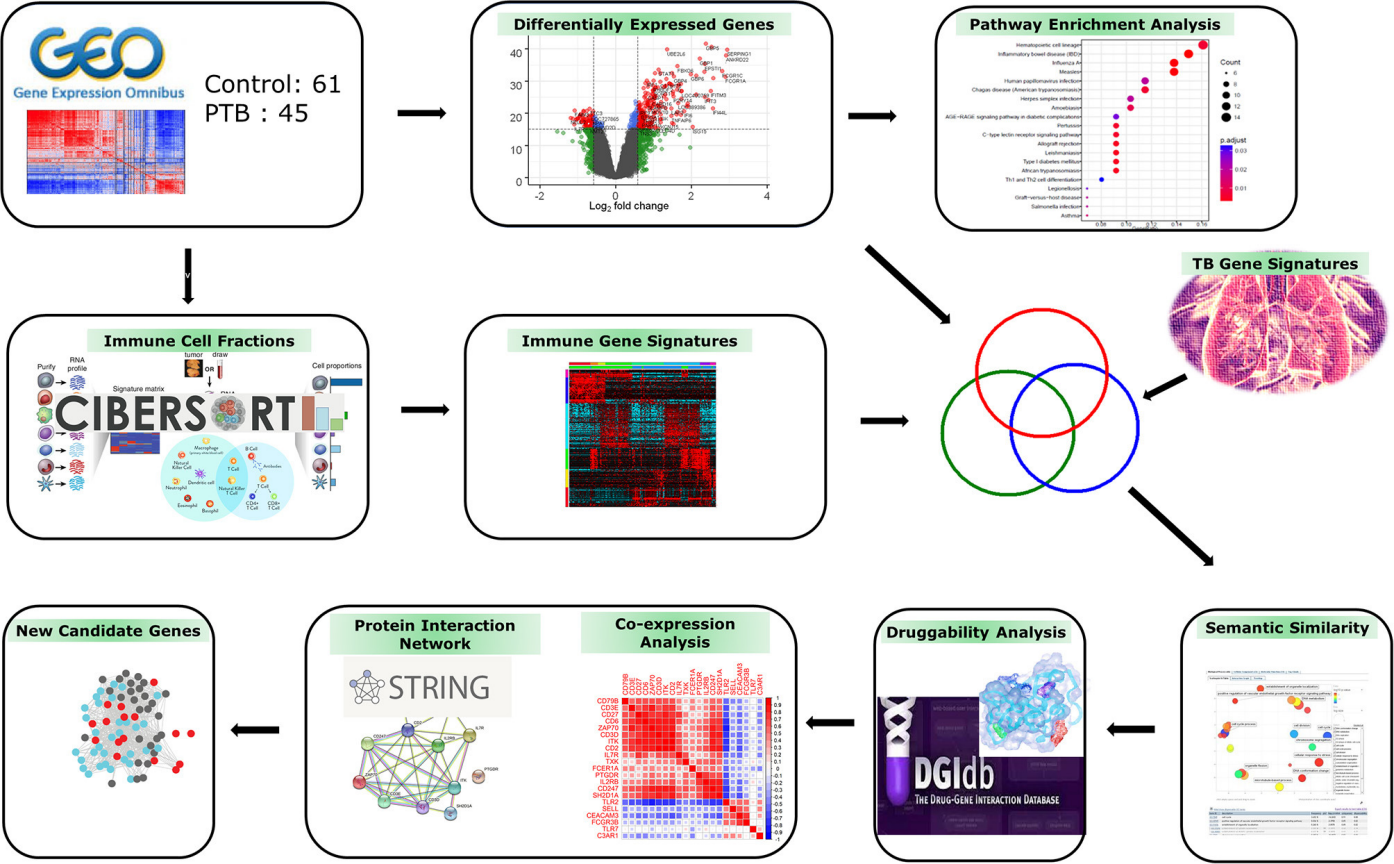
Study workflow. The gene expression profiles of patients with tuberculosis (TB) and controls were used to deconvolute immune cell fractions. Differentially expressed genes (DEGs) were mapped to functional pathways and then correlated with immune cell and TB meta-analysis gene signatures. The overlapping genes showing semantic similarity were explored by druggability and protein interaction analysis to identify novel candidate therapeutic targets/biomarkers for combating TB infection.

The genome-wide gene expression dataset (GSE83456) (13) was imported in raw format from the Gene Expression Omnibus (GEO) database (www.ncbi.nlm.nih.gov/geo). This dataset has expression profiles of 45 pulmonary TB (PTB) and 61 control blood samples generated on the Illumina Human HT-12 V4.0 expression bead chip (Illumina, Inc, USA). The detailed sample information is given in the Supplementary Material Table 1. Figure 1 depicts the overall work design employed in the current research analysis.

### Global Data Preprocessing and Screening of Differentially Expressed Genes

R/Bioconductor packages were used to analyze microarray gene expression data. Raw data was fed into the R package *limma* (14) for the standardization and noise reduction of the probe data, and the raw signal levels for each probe set were standardized. The Quantile method was used to normalize the microarray datasets. The t-statistic was used to detect statistically significant differentially expressed genes (DEGs) between the PTB and control samples. To eliminate false positives, the Benjamini and Hochberg false discovery rate (FDR) with *p* < 0.05 was used as a cut-off point for gene data. Thereafter, probes were matched to Entrez Gene IDs, and duplicates (with the highest fold change difference) and unmatched transcripts were filtered out. In the final stage, all the DEGs were classified as up- and downregulated genes based on the fold change threshold (FC ≥ |1.5|).

### Identification of Immune Cell Composition From Gene Expression Profiles

The fractions of 22 immune cell types in the PTB transcriptome profile were estimated using the CIBERSORT algorithm (15). This program employs linear support vector (SVR) regression to perform feature selection and to deconvolve the cell mixture from the gene expression profile. In this study, gene expression profiles of PTB and control samples were fed into the CIBERSORT algorithm where the algorithm converts the gene expression matrix into the immune cell-matrix and applies the filtering criteria of 1,000 permutations and significant *p* value set at ≤ 0.05.

### MTB Meta-Profiling Genetic Signatures

We obtained 380 genetic signatures identified from the modular analysis and meta-profiling of 16 publicly available gene expression datasets (16) (Supplementary Material Table 2). We compared the overlapping genes between these 380 *TB* genetic signatures, DEGs, and genes associated with immune cell populations for downstream analysis.

### Identification of Immune Pathways From Gene Expression Profiles

The functional enrichment analysis of the DEGs was performed using g:Profiler (17), a webserver to interpret the function of gene lists (https://biit.cs.ut.ee/gprofiler/gost). This server matches a queried gene list to established functional data sources and uncovers gene ontologies as well as pathway terms that are significantly enriched at q ≤ 0.05. Immune-related pathways were screened from the functional enrichment list. The DEGs which are contributing to immune-associated pathways were mapped to the known signature of TB and immune signature genes in the CIBERSORT to identify unreported genes in TB.

### Identification of Semantic Similarity

Using encoded evidence in the Gene Ontology (GO) hierarchy, the functional similarity between unreported genes and known TB signatures is assessed. In this study, we used Wang's similarity metric to compare the biological process (BP) hierarchy. To quantify the semantic similarity between gene pairs, we used the R tool GoSemSim (18).

We employed Resnik's measure of Best-Match Average (BMA) method, which combines the semantic relationship scores of numerous GO terms and produces the average of all maximal similarities in each row and column because a gene can be annotated by many GO terms (19). Following that, gene pairs were selected based on a semantic score of ≤ 0.5, with a larger score indicating a stronger relationship. The following formula was used to calculate the semantic similarity among gene pairs:


(1) $$\begin{array}{c} S_{GO}(A,B)=\frac{\sum_{t\in T_{A}\cap^{\;}T_{B}}\left(S_{A}(t)+S_{B}(t)\right)}{\sum_{t\in T_{A}}S_{A}(t)+\sum_{t\in T_{B}}S_{B}(t)} \end{array}$$

where *T*_*A*_ designates the contribution of *t* ∈ *T*_*A*_ term to the semantics of A based on the relative positions of t and A in the graph, and *S*_*A*_(*t*) implies the role of *t* ∈ *T*_*A*_ term to the semantics of A.

### Druggability Analysis

The Drug–Gene Interaction Database (DGIdb) (20) was used to assess the druggability of the genes. DGIdb is a central resource for drug-gene interaction data and the potential druggability of each query gene based on different databases. We included approved drugs, antineoplastic drugs, and Immunotherapeutic drug interactions filters and in advance filters, we selected 9 Disease-Agnositic sources databases, 43 gene categories, and 31 interaction types. We used drug target interaction with interactions score ≥0.03 to search the DEGs, which could act as potential drug target genes for MTB.

### Correlation Among the Druggable Genes

The correlation between the druggable genes in PTB was investigated using Pearson's correlation method. The correlation (r) between each pair of gene matrices was ranked using Pearson's correlation coefficient (PCC). The formula used for computing the PCC existing between two genes is given below.


(2) $$PCC\;(r)=\frac{\sum_{i=1}^{n}\left(x_{i}-\bar{x})\;(y_{i}-\bar{y}\right)}{\sqrt{\sum_{i=1}^{n}{\left(x_{i}-\bar{x}\right)}^{2}}\sqrt{\sum_{i=1}^{n}{\left(y_{i}-\bar{y}\right)}^{2}}}$$

where $\bar{x}$ and ȳ are the average of sample's gene expression signal in PTB of the two genes, respectively. The gene co-expression was confirmed using the Search Tool for the Retrieval of Interacting Genes (STRING) (21), an online protein interaction database, with high confidence interaction score of ≥0.7.

### Real Time-PCR (RT-PCR) Validation of Druggable Genes

In order to verify our bioinformatics findings, we validated the expression of 9 druggable genes by the RT-PCR method. In brief, the RNA collected from THP-1 cell lines infected with the MTB strain (H37Rv) was collected after the post-incubation period as previously described (22). In brief, total RNA was reverse transcribed as complementary DNA (cDNA) and then amplified by the RT-PCR method using gene-specific oligonucleotide primers. The relative expression level of potentially druggable genes between the control and test cell lines was estimated by the 2^−ΔΔCT^ formula after normalizing their expression levels with the GAPDH internal reference gene. A *p* < 0.05 under the standard two-tailed *t*-test was considered a significant value.

## Results

### Immune Cell Proportion Analysis of PTB Gene Expression Profile

The immune cell proportion landscape of PTB is not yet fully revealed, particularly in low abundant cell subpopulations. In this study, the CIBERSORT algorithm has identified the enrichment of genes associated with 10 types of adaptive immune cells like B cells naive, plasma cells, T follicular helper cells, CD8^+^ T cells, resting memory CD4^+^ T cells, T cells, CD4^+^ memory T cells activated, memory B cells, naive CD4^+^ T cells, regulatory T cells (Tregs), and Gamma-delta (γδ) T cells. On the other hand, DEGs associated with 12 innate immune cell type categories were NK cells resting, macrophages M2, monocytes, macrophages M1, macrophages M0, resting dendritic cells, eosinophils, dendritic cells activated, mast cells resting, NK cells activated, mast cells activated, and neutrophils were also found be enriched. The immune cell proportions of adaptive immune cells and innate immune cells are represented in Supplementary Material Figure 1.

**Figure 2:**
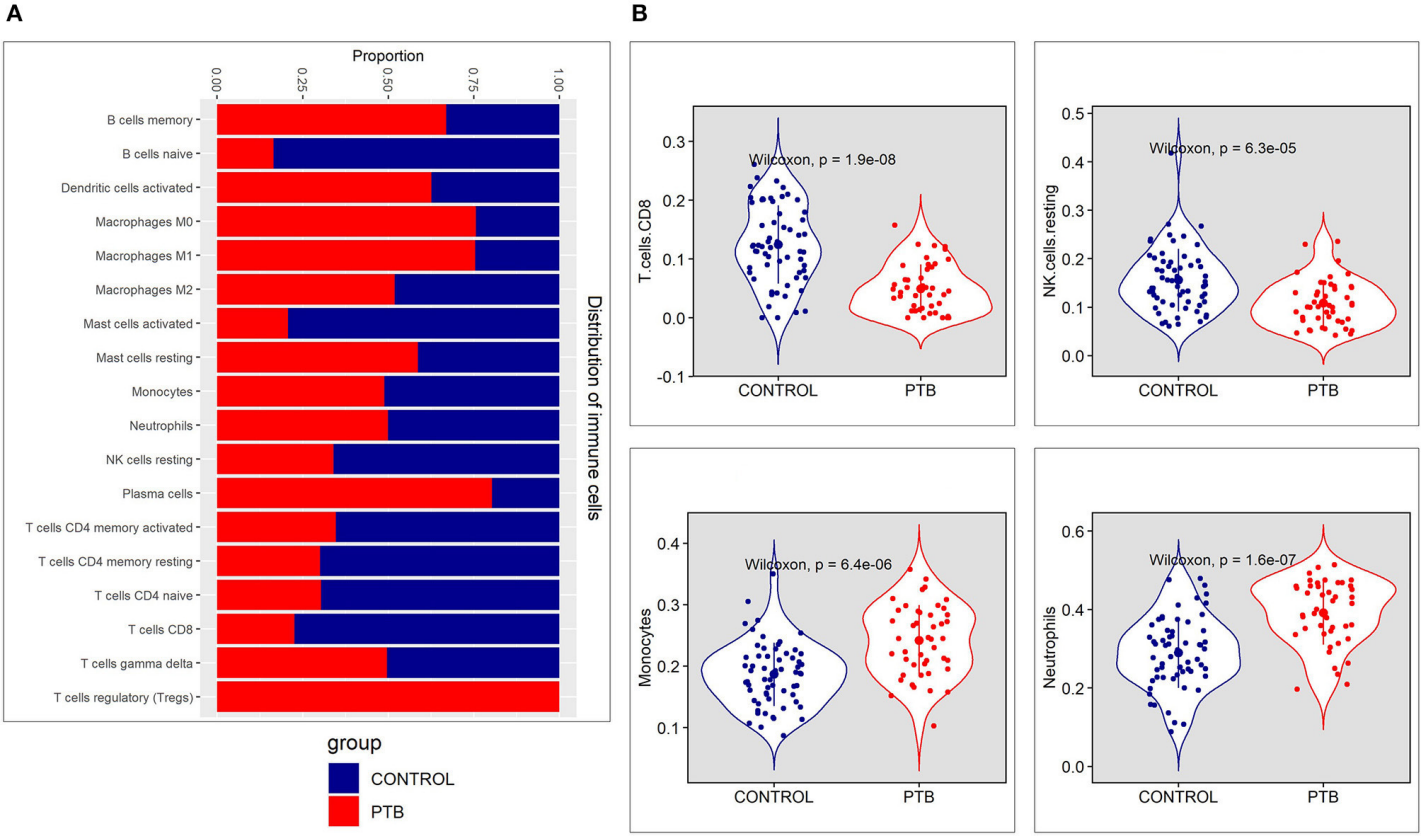
The immune cell proportion landscape between pulmonary TB (PTB) and controls. **(A)** The relative proportion of immune cell subpopulations in GSE83456 dataset. **(B)** The difference of immune infiltration between PTB and normal controls (the control group was marked in blue color and the PTB group was marked in red color. *P* < 0.05 were considered as statistically significant).

The genes associated with four immune cells; NK cells activated, T follicular helper cells, dendritic cells resting, and eosinophils, were not significantly enriched in both groups. The proportion plot of the enriched immune cell types is represented in Figure 2A. We observed higher relative proportion of genes enriched for cell types like CD8^+^ T cells (*P* = 1.9 × 10^−8^), NK cells resting (*P* = 6.3 × 10^−5^), monocytes (*P* = 6.4 × 10^−6^), and neutrophils (*P* = 1.6 × 10^−7^) in the PTB samples compared to the control samples (Figure 2B, Supplementary Material Figure 1). Among the 4 cell types with a higher relative proportion of enriched genes from DEGs, the genes of CD8^+^ T cells and NK resting cells were found to be downregulated in PTB when compared to the control samples. On other hand, monocytes and neutrophil-associated genes were highly active in PTB when compared to control samples.

### Identification of DEGs From Gene Expression Profile

**Figure 3:**
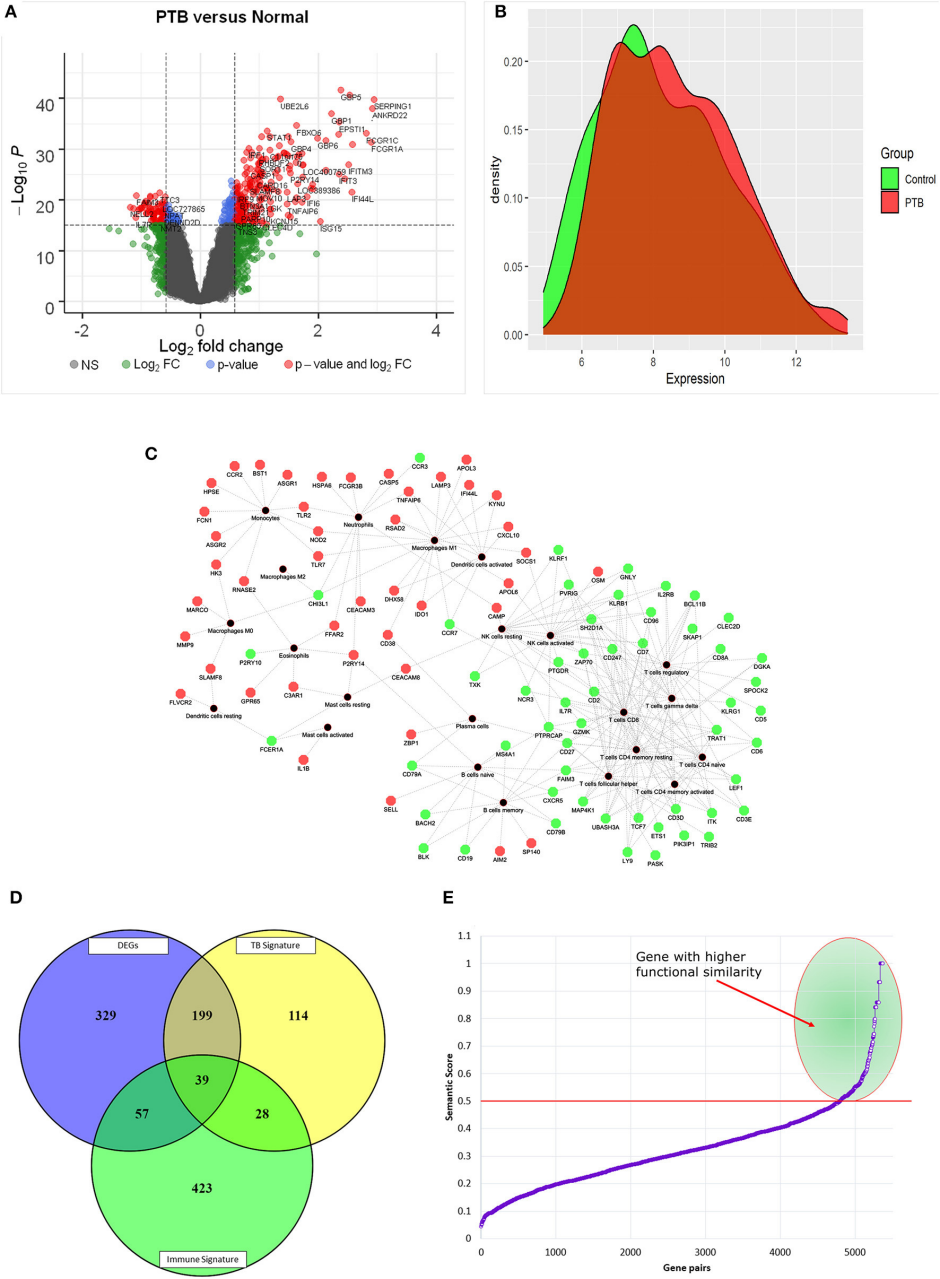
Graphical distribution of differentially expressed genes. **(A)** Volcano plot representing the distribution of fold change and p-value significance. **(B)** The distribution mean intensity of differentially expressed genes in the PTB and control samples. **(C)** Red and green nodes represent up and downregulation of genes and black nodes are the immune cell types. **(D)** The Venn diagram represents the overlap of DEGs with immune and TB signatures. **(E)** Semantic similarity of pairs of genes between differentially expressed immune signatures and TB signatures. The selected gene pairs with higher functional similarity (≥0.5) are highlighted in green color.

The standardized gene expression data of “PTB vs. controls” was used to identify the differentially expressed genes. The volcano plot representing the distribution of fold change and the significant *p*-value is given in Figure 3A. The PTB vs. control group analysis revealed 624 DEGs (FC |1.5|, adj *p*-value of 0.05), with 393 upregulated and 231 downregulated genes. The top 10 DEGs obtained from PTB vs controls are given in Table 1. The mean distribution of intensity of differentially expressed genes in PTB and control samples is represented in Figure 3B.

**Table 1:** The top 10 differentially expressed gene list in pulmonary tuberculosis (PTB).

**Symbol**	**FC**	**Gene name**	**Adj *P* value**
*SERPING1*	7.75	Serpin family G member 1	2.08E−36
*ANKRD22*	7.58	Ankyrin repeat domain 22	9.83E−35
*FCGR1A*	7.51	Fc fragment of IgG receptor Ia	1.00E−28
*FCGR1C*	7.04	Fc fragment of IgG receptor Ic, pseudogene	3.79E−30
*FCGR1B*	6.02	Fc fragment of IgG receptor Ib	2.75E−28
*LRRN3*	−2.91	Leucine rich repeat neuronal 3	4.35E−13
*FCGBP*	−2.60	Fc fragment of IgG binding protein	1.03E−12
*NELL2*	−2.27	Neural EGFL like 2	6.95E−17
*GZMK*	−2.20	Granzyme K	2.19E−10
*CCR7*	−2.19	C-C motif chemokine receptor 7	1.15E−16

### Functional Enrichment Analysis of DEGs

The differentially expressed genes enriched using g:Profiler with the statistical significance of q value ≤ 0.05, generated 309 ontologies of Biological Process (BP), 17 ontologies of Molecular Function (MF), 42 ontologies of Cellular Component (CC), and 85 terms in pathways (Supplementary Material Table 3). Overall, the enrichment analysis has shown the overlap with immune-related ontologies and pathways. We pooled immune-related pathways from enrichment terms to check how DEGs affect the immune system pathways. We observed the upregulation of pathways such as interferon signaling (q = 9.16 × 10^−27^), cytokine signaling in the immune system (q = 2.34 × 10^−21^), neutrophil degranulation (q = 3.13 × 10^−11^), viral genome replication (q = 2.47 × 10^−7^), and response to biotic stimulus, etc. (Supplementary Material Figure S1). On the other hand, pathways like T-cell antigen receptor signaling, antigen receptor-mediated signaling, NF-kappa B signaling, T cell activation, T cell receptor signaling, leukocyte differentiation, leukocyte activation, alpha-beta T cell activation, and T cell differentiation, were downregulated (Supplementary Material Figure S1). Overall, our functional enrichment analysis points to a major downregulation of T cell-related ontologies and pathways.

### Mapping DEGs to Immune Cell Proportions in PTB

Here we investigated the genes overlapping between the CIBERSORT signature and DEGs. There are about 96 DEGs (Figure 3C) contributing to different immune cell types (Supplementary Material Table 4). Interestingly, we found that 31.25% of DEGs were contributing to the immune cell type “CD8^+^ T cells.” We also observed that all those genes contributing to the “CD8^+^ T cells” were downregulated in PTB samples, as shown in Figure 3C. The findings from the mapping of DEGs to immune cell proportions are consistent with functional enrichment analysis, where T cell-related pathways have shown major dysregulation.

### Comparison of DEGs, Immune Cell Signatures With TB Meta-Analysis Signatures

The differentially expressed immune signatures in the sample of patients with TB were compared with the known signatures of TB (Supplementary Material Table 4). Here, we observed 39 (40.6%) differentially expressed immune signatures overlapping with TB meta-analysis gene signatures and 57 novel genes (59.3%) contributing to the immune cell proportion (Figure 3D). Further, semantic similarity (functional association) of these 57 novel genes with 39 overlapping with TB signatures was performed to identify the most predominant genes. The semantic similarity score of ≥0.5 among the gene pairs was considered as a highly significant score implying a stronger association. The semantic similarity of 45/57 genes has shown a stronger functional association with overlapping with TB signatures **(Figure 3E)**. Again, it is important to pinpoint that 20 out of 45 genes (44%) were contributing to the immune cell type “CD8+ T cells.”

### Druggability and Co-expression Analysis

**Figure 4:**
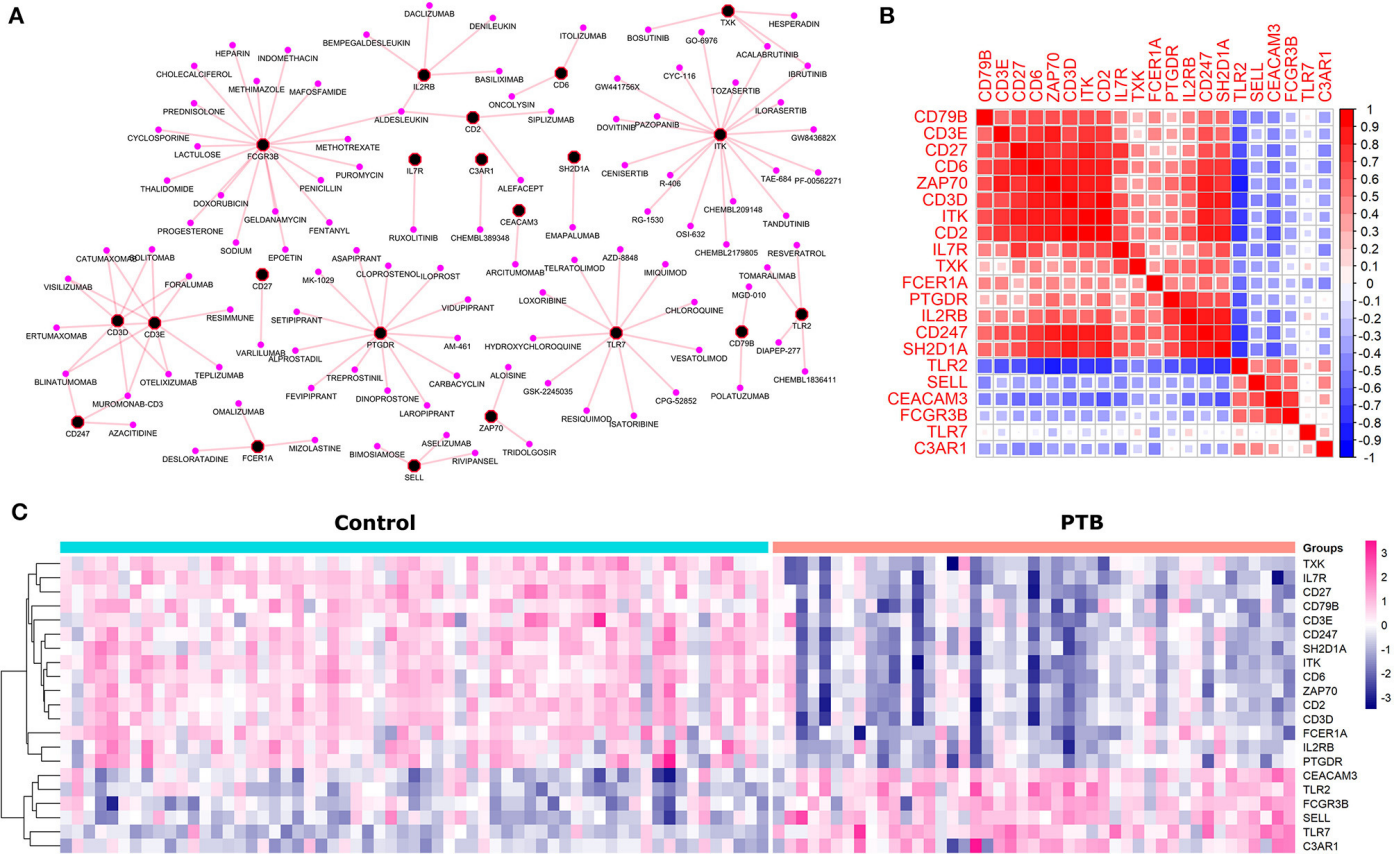
The druggable targets with their expression, interaction and co-expression **(A)** The network depicts the drug-target interaction where black and pink nodes represent target and drugs respectively. **(B)** The co-expression (a similar pattern of gene expression) among the druggable genes where red and blue represent the positive and negative correlation. **(C)** The pattern of gene expression of druggable genes in control and PTB patient samples clearly depicts a distinction between the control and PTB groups.

Druggability analysis was performed on the 45 genes that had shown higher functional similarity to the known TB signature. We found that 21 druggable genes (Supplementary Material Figure S2) (46%) with an interaction score ≥ 0.03, were enriched against terms like an antibody, binder, inhibitor, antagonist, agonist, modulator, and activator. Of all the druggable genes, *ITK* and *FCGR3B* genes were observed to have the highest number of drug interactions (19 drugs) followed by *PTGDR* (13 drugs), *TLR7* (12 drugs), and *CD3E* (10 drugs). The drug-target interaction network is represented in Figure 4A. Next, we checked the association of druggable targets to the immune cell types. Among the 21 druggable targets, 48% were contributing to the immune cell type “CD8^+^ T cells” followed by monocytes (9%), naïve B cells (9%), and neutrophils (9%) (Figure 4B). Interestingly, the expression patterns of these 21 druggable genes have shown a clear distinction in PTB when compared to control samples (Figure 4C).

**Figure 5:**
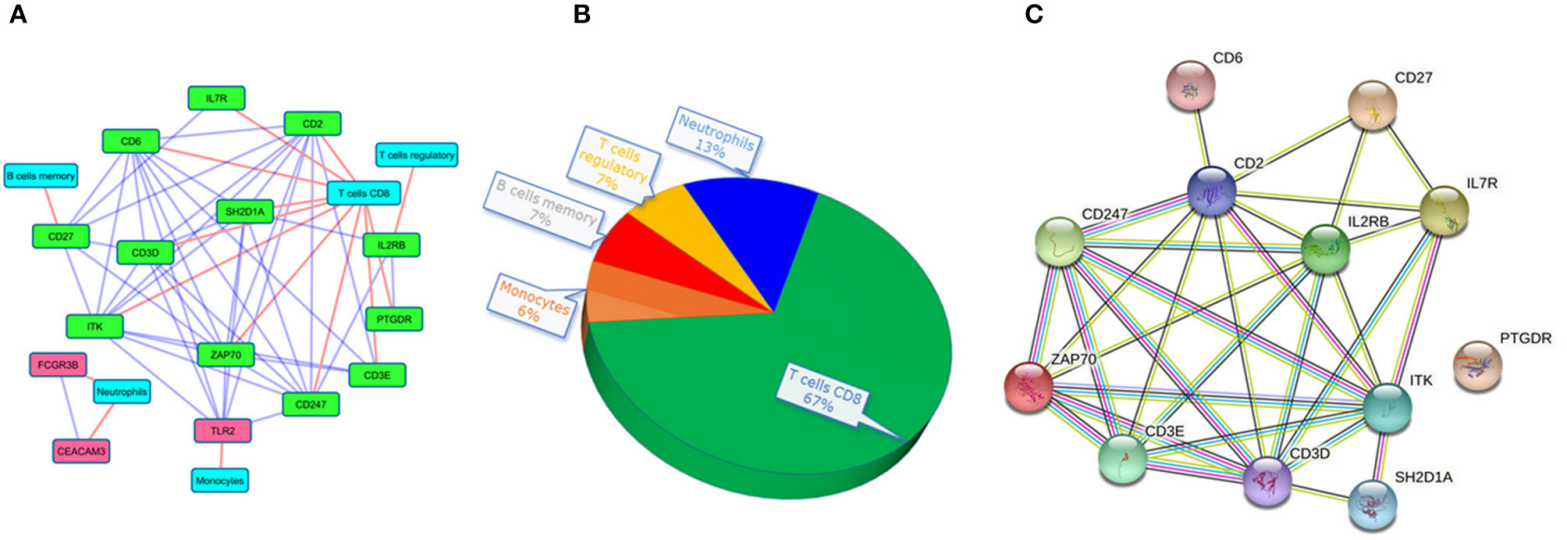
The interaction networks of druggable targets. **(A)** co-expressed network of druggable targets where red and green colored nodes represent up and downregulated genes. The red edge represents the association of targets to immune cells and the blue edges depict the interaction among the target genes. **(B)** The pie chart represents the composition of immune cell types with the co-expressed druggable targets. **(C)** The interaction of the co-expressed druggable targets in the STRING database.

To check the correlation of these 21 druggable targets in patients with PTB, we performed Pearson's correlation analysis (Figure 4B). The correlation analysis performed between druggable targets in PTB samples resulted in 15 genes (*ITK, CD2, CD6, CD247, CD27, CD3D, ZAP70, SH2D1A, IL2RB, CEACAM3, IL7R, TLR2, CD3E, PTGDR, FCGR3B*) with higher Pearson correlation coefficient (r^2^ ≥ |0.7|). The co-expressed genes are shown in Table 2. Among the 15 co-expressed genes, 12 and 3 were down and upregulated respectively (Figure 5A). Upregulated genes were contributing to the immune cell types “Monocytes” (*TLR2*) and Neutrophils (*FCGR3B* and *CEACAM3*). Again, 10 downregulated genes were contributing to “T cells CD8” and 1 each for “T cells regulatory” and “B cells memory” (Figure 5B).

**Table 2:** List of co-expressed genes and the druggable targets.

**Gene**	**Immune cells**	**Regulation**	**Co-expressed genes**	**PCC range**
*ITK*	CD8^+^ T cells	DOWN	*CD2, CD3D, ZAP70, CD247, SH2D1A, CD3E, TLR2, CD6, CD27*	0.72–0.91
*CD2*	CD8^+^ T cells	DOWN	*CD3D, SH2D1A, ZAP70, CD247, TLR2, CD3E, ITK, CD6, CD27*	0.74–0.91
*CD6*	CD8^+^ T cells	DOWN	*ITK, ZAP70, CD2, CD3D, CD247, SH2D1A, TLR2, CD3E, CD27*	0.74–0.89
*ZAP70*	CD8^+^ T cells	DOWN	*TLR2, CD247, CD3E, SH2D1A, CD6, CD2, ITK, CD3D, CD27*	0.76–0.88
*CD247*	CD8^+^ T cells	DOWN	*SH2D1A, IL2RB, TLR2, CD2, CD3D, ITK, ZAP70, CD6*	0.79–0.87
*CD3D*	CD8^+^ T cells	DOWN	*CD247, SH2D1A, ZAP70, TLR2, CD2, ITK, CD6, CD27*	0.72–0.90
*SH2D1A*	CD8^+^ T cells	DOWN	*IL2RB, TLR2, CD2, CD247, CD3D, ITK, CD6, ZAP70*	0.73–0.88
*TLR2*	Monocytes	UP	*ZAP70, CD247, CD6, CD2, SH2D1A, ITK, CD3D*	0.72–0.82
*CD27*	B cells memory	DOWN	*CD6, ITK, CD3D, CD2, ZAP70, IL7R*	0.71–0.85
*CD3E*	CD8^+^ T cells	DOWN	*ZAP70, ITK, CD6, CD2*	0.74–0.80
*IL2RB*	T cells regulatory	DOWN	*SH2D1A, CD247, PTGDR*	0.79–0.82
*CEACAM3*	Neutrophils	UP	*FCGR3B*	0.71
*IL7R*	CD8^+^ T cells	DOWN	*CD27*	0.71
*PTGDR*	CD8^+^ T cells	DOWN	*IL2RB*	0.79
*FCGR3B*	Neutrophils	UP	*CEACAM3*	0.71

*#PCC, Pearson correlation coefficient*.

Interestingly, we noticed a cluster of 12 co-expressed genes *ITK, CD2, CD6, ZAP70, CD247, CD3D, SH2D1A, CD27, CD3E, IL2RB, IL7R*, and *PTGDR*. To validate the co-expression among the 12 downregulated genes we queried them in the STRING database with a high confidence score of ≥ 0.7. The STRING database identified strong interaction among the 11 genes except for PTGDR (Figure 5C). Co-expression and protein interaction network from the STRING database has shown the mutual influence of the 11 genes in the expression and functional activities. Nine genes (expect *CD27, PTGDR*, and *IL2RB*) were contributing to “CD8^+^ T cells.” All the genes were predicted to be targeted by different drug molecules, most of which are monoclonal antibodies (Table 3). Hence, the integrated analysis has depicted predominant deregulation of “CD8^+^ T cells” as the key genetic signatures for active PTB.

**Table 3:** The list of drugs shows direct interaction with 9 genes associated with CD+T cell functioning.

**Gene**	**Drug**	**Interaction type and** **directionality**	**Sources**	**PMIDs**	**Query** **Score**	**Interaction** Score^*^
ITK	CHEMBL2179805	–	DTC	(23)	8.77	6.71
CD2	ALEFACEPT	Inhibitor (inhibitory)	TdgClinicalTrial	(24–26)	21.92	106.32
			ChemblInteractions TEND	(27, 28)		
			GuideToPharmacology	(29)		
	SIPLIZUMAB	Inhibitor (inhibitory)	TdgClinicalTrial	–	13.15	63.79
			ChemblInteractions TTD			
CD6	ITOLIZUMAB	Antibody (inhibitory)	GuideToPharmacology TTD	–	8.77	63.79
	ONCOLYSIN CD6	–	ChemblInteractions	–	4.38	31.9
CD247	MUROMONAB-CD3	–	TdgClinicalTrial	(30, 31)	9.86	15.95
ZAP70	TRIDOLGOSIR	–	DTC	(32)	2.92	21.26
	ALOISINE	Inhibitor (inhibitory)	GuideToPharmacology	–	1.46	10.63
CD3D	MUROMONAB-CD3	Inhibitor (inhibitory)	TdgClinicalTrial	(30, 31)	13.15	9.11
			ChemblInteractions			
	BLINATUMOMAB	Activator (activating)	TdgClinicalTrial	–	5.85	6.08
			ChemblInteractions			
SH2D1A	EMAPALUMAB	–	PharmGKB FDA	–	1.1	15.95
CD3E	MUROMONAB-CD3	Binder, inhibitor (inhibitory), antibody (inhibitory)	TdgClinicalTrial	(33, 34)	26.31	12.76
			ChemblInteractions TEND			
GuideToPharmacology TTD	OTELIXIZUMAB	Antibody (inhibitory)	TdgClinicalTrial	–	8.77	6.38
			ChemblInteractions			
			GuideToPharmacology			
IL7R	RUXOLITINIB	–	CGI	(35, 36)	1.1	15.95

* *Drug molecules showing >5 interaction score is shown here*.

### RT-PCR Validation of Druggable Genes

The real-time PCR gene expression results showed that the relative expression levels of 9 potential druggable genes (*ITK, CD2, CD6, CD247, ZAP70, CD3D, SH2D1A, CD3E, and IL7R*) were consistent with the findings of microarray hybridization. All the genes were differentially expressed between treated and untreated cell lines (*p* ≤ 0.01). These results confirm the dysregulated “CD8^+^ T cell signaling” plays important role in establishing TB infection (Supplementary Material Figure S3).

## Discussion

Host genetic factors are known to play an important role in regulating the initial TB infection and determining the disease progression in the lungs (37). Genome-wide association studies have underlined the relevance of numerous polymorphisms in immune response-related genes in contributing to susceptibility or resistance to TB (38). However, polymorphism studies were unable to provide full insight into the complex molecular crosstalk between thousands of host genes involved in innate and adaptive immune responses. In this context, high throughput transcriptome approaches have shown great promise in dissecting the host-pathogen interactions thereby helping to develop a novel vaccine and therapeutic targets for several infectious diseases (39–41). Therefore, we explored host immune system response through integrated systems biology approach based on immune cell subtyping and differential gene expression profiles of patients with PTB to normal controls.

The cellular and molecular background of TB-induced systemic immunological dysregulation is poorly understood. Therefore, we screened the DEGs in PTB and deciphered their contribution to immune cell proportion alterations. Traditionally, host transcriptomics studies have relied on whole blood to characterize TB gene signatures by aggregating transcriptomic signals from many different cell types but were unable to identify specific immune cell type signatures (42). To overcome these constraints, we used a powerful computational technique called CIBERSORT to define the range of immune cell states in the blood of patients with TB. This method relies on linear support vector regression (SVR), a machine learning approach to deconvolute the gene expression signatures, known as “signature matrix” for determining the relative fraction of immune cell proportions in blood or tissues (15). The CIBERSORT method has been widely used to infer immune cell types from transcriptomics data to predict outcomes of different cancers (9, 43, 44) and infectious diseases (45–47). In this study, the CIBERSORT output identified the downregulation of “CD8^+^ T cells” in patients with PTB.

The GO functional enrichment analysis of gene expression profile revealed the upregulation of interferon signaling, cytokine signaling in the immune system, neutrophil degranulation, and response to biotic stimulus pathways. The MTB infection of primary human macrophages is shown to induce type I IFN signaling and limit the expression of IL-1β, which imparts immunity against the infection (48). Additionally, the downregulation of major pathways associated with T cells function like T-cell antigen receptor signaling, leukocyte differentiation, leukocyte activation, T cell activation, T cell differentiation, T cell receptor signaling, alpha-beta T cell activation, NF-kappa B signaling, and antigen receptor-mediated signaling pathway were noted in PTB samples.

Majority of the DEGs contributing to the immune cell type “CD8^+^ T cells” were clearly downregulated in the PTB samples indicating their potential roles in defense against TB. These cells are also known as killer or cytotoxic T lymphocytes, as they potentially destroy the infected cells by recruiting cytokines and other immune cells to the site of infection. The low abundance of blood CD8^+^ T lymphocytes may impair the effective immunity against pathogens, as they lack a sufficient cytotoxic T cells to recognize the MHC class I-restricted epitopes of MTB antigens, in the site of infection (49). A recent RNA transcriptome study used the positron emission tomography (PET) data collected from recovered patients with TB, at 4th and 24th weeks has also reported that genes associated with the overexpression of B cells and down expression of T cells and platelets confirms our findings (50). Thus, the downregulation that contribute to immune cell type is concordant with the pathway enrichment analysis findings of lower expression of T cell-related ontologies and pathways.

The druggability potential of any protein is attributed to its binding specificity with small compounds following Lipinski's rule-of-five for drug likeliness (51). Numerous bioinformatic and empirical methods which consume less time and provide faster prescreening of druggability of candidate proteins than conventional methods have been developed (52, 53). There are a variety of computational methods available, which can predict druggability and protein-binding sites by using energy dynamics to geometrical topological estimations, and from flexible to rigid proteins (54, 55).

By applying druggability and co-expression features we identified 9 CD8^+^ T cells associated genes (*ITK, CD2, CD6, CD247, ZAP70, CD3D, SH2D1A, CD3E*, and *IL7R*) as potential therapeutic targets of PTB. However, it is pivotal to carefully prioritize the drug molecules based on their mode of action, whether activator or inhibitor based on the gene expression status. For example, over-expressed genes can be targeted by inhibitory molecules, and downregulated genes can be targeted by activator molecules (56). From the above 9 genes, the therapeutic potential of ITK and IL7R has been characterized by experimental methods. ITK is a tyrosine kinase expressed on T-cells, which regulates its T-cell development and function. Human lungs with ITK deficiency impair early protection against MTB *in vivo* (57). Improving ITK signaling pathways could become an alternative approach for combating MTB infection. One study reveals the role of IL7R on T-cell immunity in human TB (58). The authors reported that patients with TB had lower IL7R concentrations and lower IL7R expression in T cells than healthy controls, indicating that patients with TB have impaired T-cell sensitivity. In addition, due to post-transcriptional processes, patients with TB had reduced amounts of IL7R in T cells. *In vitro* experiments revealed that MTB-specific T lymphocytes from patients with TB have reduced IL-7-induced STAT5 phosphorylation and IL-7-promoted cytokine production (59). The role of the remaining 7 genes (*CD2, CD6, CD247, ZAP70, CD3D, SH2D1A*, and *CD3E*) in T-cell signaling and modulation of host immune responses in mycobacterium infections is also supported (60–64).

Our results highlight the dysregulation of CD8^+^ T cells and the associated genes in PTB patients. These findings are exciting not just from the fact that CD8^+^ T cell-associated genes have the potential to act as potential therapeutic targets but prove that their role is not less important than CD4^+^ T cells in controlling MTB infection. We acknowledge that our strategy has some technical constraints. CIBERSORT was a convenient computational tool for determining infiltrating immune cell fractions, but it was still less precise than immunohistochemistry or flow cytometry, which could lead to inaccuracies in immune cell fractions. However, to overcome this limitation to some extent, we have linked gene expression profiles of immune signatures followed by functional enrichment, semantic similarity, druggability, and co-expression among the identified key signatures.

## Conclusion

In this study, by coupling computational deconvolution algorithms and high throughput blood transcriptomics data, we identified the difference in T-cell-related immune cell populations among patients with PTB. The functional enrichment of 624 DEGs (393 over-expressed and 231 under-expressed) identified in the blood transcriptome of PTB patients revealed the major dysregulation of T cell-related ontologies and pathways. By linking DEGs against immune cell populations and TB gene signature, this study identified 9 CD8^+^ T cells associated genes (*ITK, CD2, CD6, CD247, ZAP70, CD3D, SH2D1A, CD3E, and IL7R*) as potential therapeutic targets of PTB. The expression levels of these 9 genes in MTB infection in cell lines were assessed by RT-PCR-based expression assay, confirming the experimental validation. However, further *in vitro* and *in vivo* studies are needed to establish the role of these genes in PTB infection, progression, and treatment.

## Data Availability Statement

Publicly available datasets were analyzed in this study. This data can be found here: http://www.ncbi.nlm.nih.gov/geo; GSE83456.

## Author Contributions

FA, BB, VV, and NS: conceptualization. FA, HA, SP, and BB: data curation. FA and BB: formal analysis. FA: funding acquisition and project administration. FA, NZ, SP, and BB: methodology. BB and SP: software and visualization. FA, NS, and BB: supervision. BB: validation. FA, NZ, HA, SP, PS, VV, RE, NS, and BB: writing original draft and editing. All authors contributed to the article and approved the submitted version.

## Funding

The authors extend their appreciation to the Deputyship for Research and Innovation, Ministry of Education in Saudi Arabia for funding this research work through the project number IFPRC-063-247-2020 and King Abdulaziz University, DSR, Jeddah, Saudi Arabia.

## Conflict of Interest

The authors declare that the research was conducted in the absence of any commercial or financial relationships that could be construed as a potential conflict of interest.

## Publisher's Note

All claims expressed in this article are solely those of the authors and do not necessarily represent those of their affiliated organizations, or those of the publisher, the editors and the reviewers. Any product that may be evaluated in this article, or claim that may be made by its manufacturer, is not guaranteed or endorsed by the publisher.
