# Supplementary material for: Transcriptome-Based Molecular Networks Uncovered Interplay Between Druggable Genes of CD8+ T Cells and Changes in Immune Cell Landscape in Patients With Pulmonary Tuberculosis
Source: Front Med (Lausanne). 2022 Feb 7;8:812857. doi: 10.3389/fmed.2021.812857 (PMC8859411; doi:10.3389/fmed.2021.812857)
Supplement: Supplementary file 2 [file Table_2.DOCX]

**Supplementary File 2**

**Adaptive immune cells**


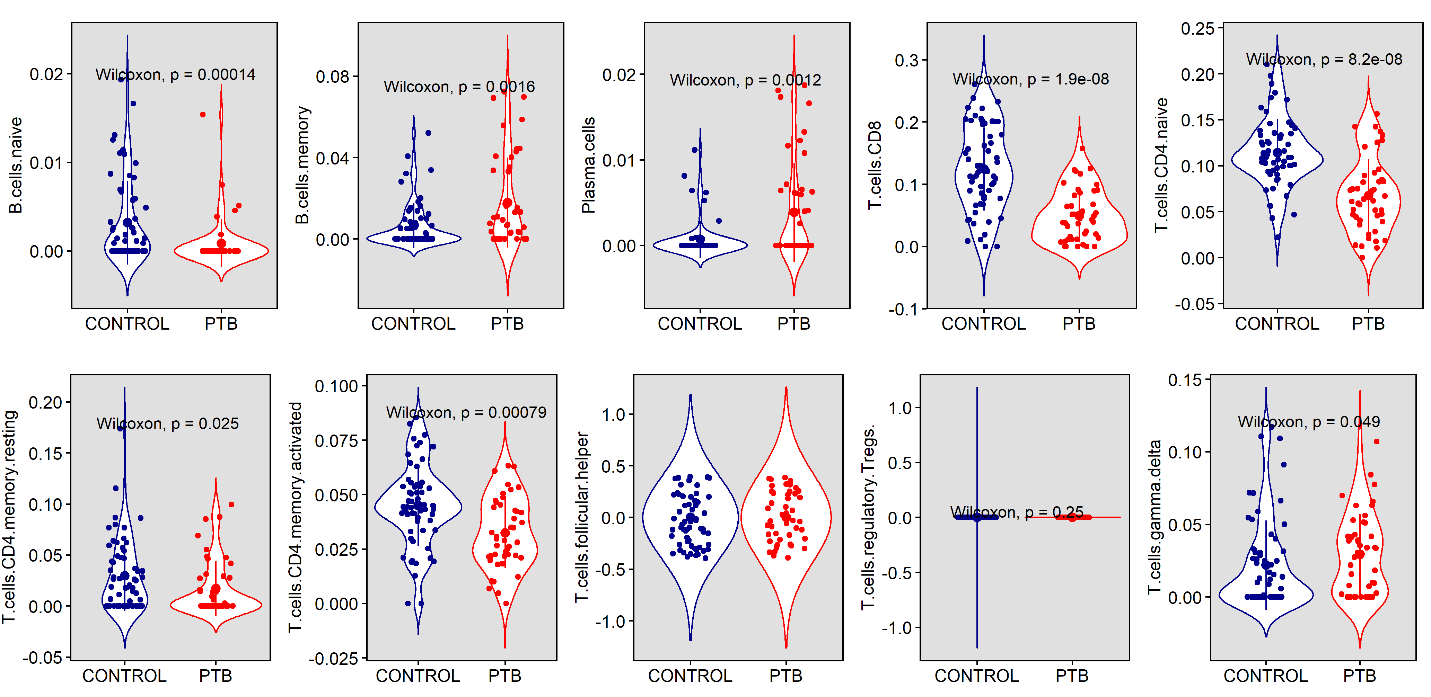


**Innate immune cells**


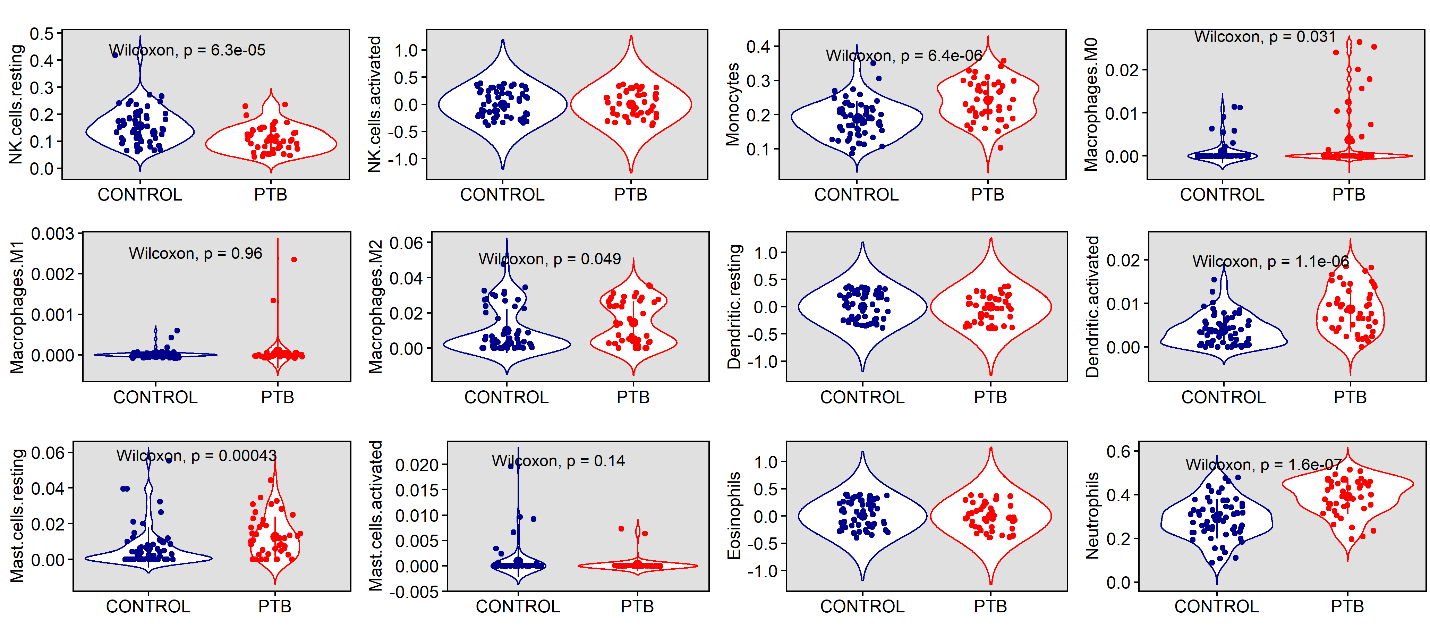


Fig 1. The distribution of immune cell types classified as adaptive and innate immune cells
