# Supplementary material for: Transcriptome-Based Molecular Networks Uncovered Interplay Between Druggable Genes of CD8+ T Cells and Changes in Immune Cell Landscape in Patients With Pulmonary Tuberculosis
Source: Front Med (Lausanne). 2022 Feb 7;8:812857. doi: 10.3389/fmed.2021.812857 (PMC8859411; doi:10.3389/fmed.2021.812857)
Supplement: Supplementary file 3 [file Table_3.DOCX]

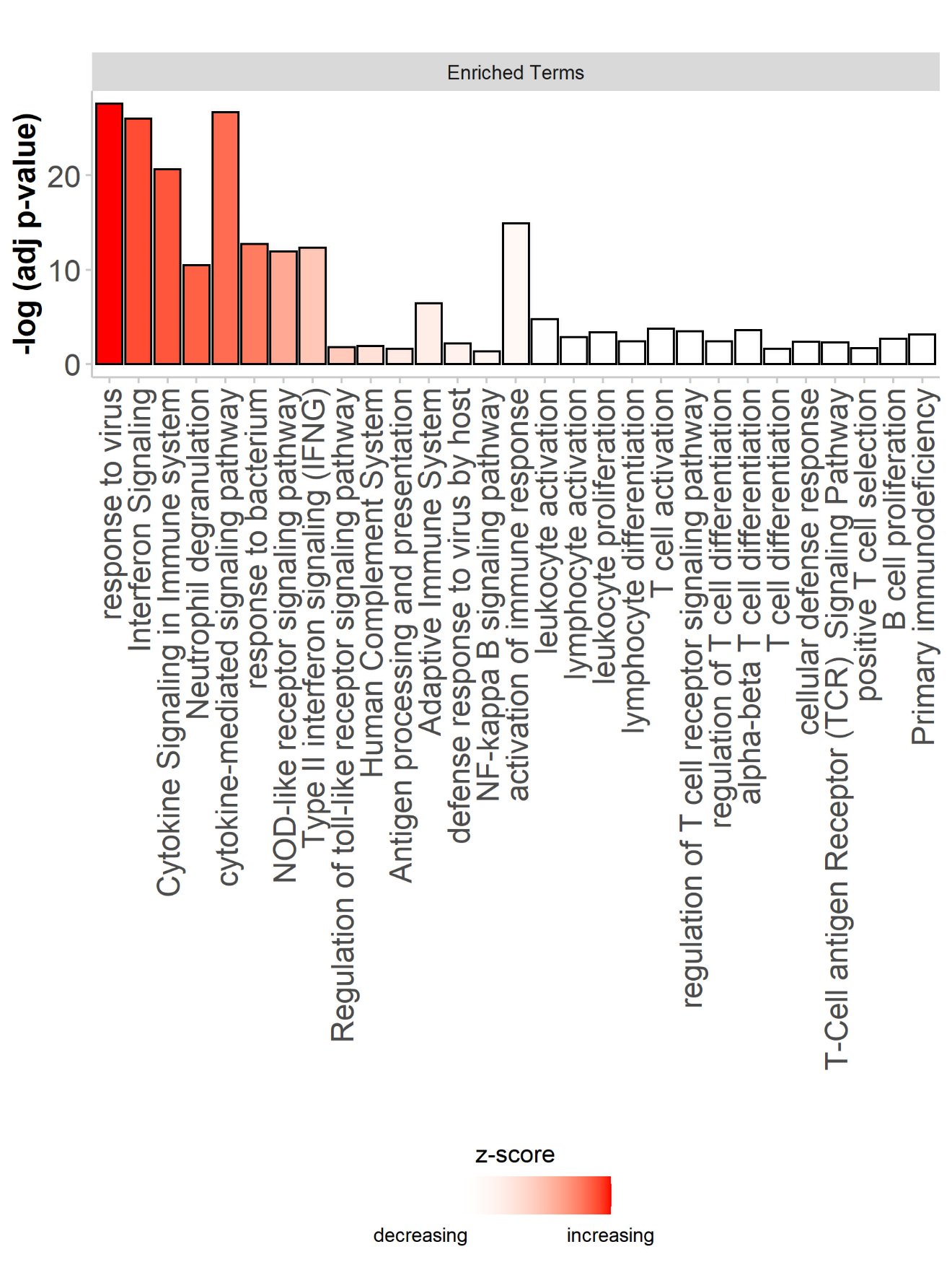


**Supplementary Figure S1:** **Overview of functional enrichment**. The gradient of the red color represents activation and deregulation of pathways.


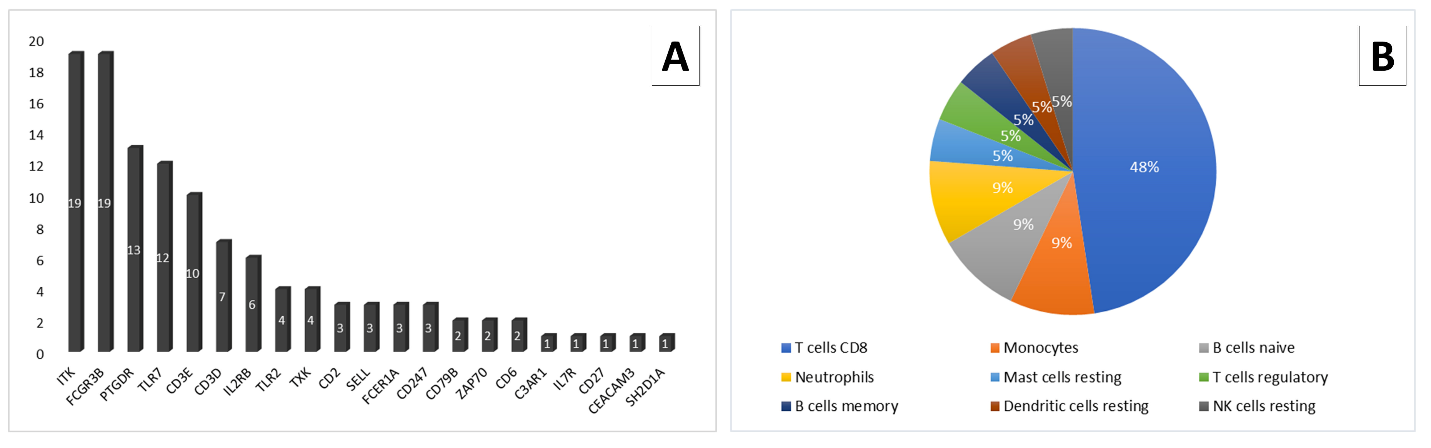


**Supplementary Figure S2 : The druggability analysis performed using DGI database to identify druggable genome in the target.** A) Frequency of drug interactions per target molecules where x axis and y axis represent targets and their corresponding counts respectively. B) The distribution of druggable targets in the immune cell types.





**Supplementary Figure S3:** Bar diagram showing RT PCR fold changes of 9 CD8^+^T cell genes in M.tuberculosis infected and control cell lines. P values are <0.05
